# Disruption of Splenic Lymphoid Tissue and Plasmacytosis in Canine Visceral Leishmaniasis: Changes in Homing and Survival of Plasma Cells

**DOI:** 10.1371/journal.pone.0156733

**Published:** 2016-05-31

**Authors:** Joselli Silva-O’Hare, Isabela Silva de Oliveira, Thaís Klevorn, Valter A. Almeida, Geraldo G. S. Oliveira, Ajax M. Atta, Luiz Antonio R. de Freitas, Washington L. C. dos-Santos

**Affiliations:** 1 Fundação Oswaldo Cruz - BA, Centro de Pesquisas Gonçalo Moniz, Salvador, BA, Brazil; 2 Faculdade de Farmácia, Universidade Federal da Bahia, Salvador, BA, Brazil; 3 Faculdade de Medicina da Bahia, Universidade Federal da Bahia, Salvador, BA, Brazil; University of Miami, UNITED STATES

## Abstract

Visceral leishmaniasis (VL) is a disease caused by *Leishmania infantum*, which is transmitted by phlebotomine sandflies. Dogs are the main urban reservoir of this parasite and the disease presents similar characteristics in both humans and dogs. In this paper, we investigated the potential pathways involved in plasma cell replacement of normal cell populations in the spleen, with respect to disease severity in dogs from an endemic area for visceral leishmaniasis. To this end, canine spleen samples were grouped into three categories: TYPE1SC- (non-infected dogs or without active infection with organized white pulp), TYPE1SC+ (infected dogs with organized white pulp) or TYPE3SC+ (infected animals with disorganized white pulp). We analyzed the distribution of different plasma cell isotypes (IgA, IgG and IgM) in the spleen. The expression of cytokines and chemokines involved in plasma cell homing and survival were assessed by real time RT-PCR. Polyclonal B cell activation and hypergammaglobulinemia were also evaluated. The proportion of animals with moderate or intense plasmacytosis was higher in the TYPE3SC+ group than in the other groups (Fisher test, P<0.05). This was mainly due to a higher density of IgG+ plasma cells in the red pulp of this group. The albumin/globulin ratio was lower in the TYPE3SC+ animals than in the TYPE1SC- or TYPE1SC+ animals, which evidences VL-associated dysproteinemia. Interestingly, TYPE3SC+ animals showed increased expression of the BAFF and APRIL cytokines, as well as chemokine CXCL12. Aberrant expression of BAFF, APRIL and CXCL12, together with amplified extrafollicular B cell activation, lead to plasma cell homing and the extended survival of these cells in the splenic red pulp compartment. These changes in the distribution of immunocompetent cells in the spleen may contribute to the progression of VL, and impair the spleen’s ability to protect against blood borne pathogens.

## Introduction

The spleen is a secondary lymphoid organ involved in the surveillance against bloodborne pathogens [1]. The protection provided by the spleen is dependent upon compartmentalized microenvironments suitable for antigen trapping, in addition to cell interactions leading to the development and maintenance of the immune response against circulating pathogens [1,2]. In humans, the absence of the spleen secondary to surgery or hematological diseases is associated with an increased risk of sepsis, as well as overwhelming infections by viruses, bacteria, protozoa and fungi [3,4]. In dogs, splenectomy is associated with an increased susceptibility to severe and disseminated infections by *Bartonella canis*, *Babesia canis* and *Mycoplasma haemocanis* [5,6,7].

The splenic microenvironment may change in the course of infections by a variety of pathogens, including viruses, *Plasmodium spp*. and *Leishmania* [8,9,10,11]. Throughout the course of visceral leishmaniasis (VL), the spleen initially develops lymphoid tissue hyperplasia, which is then followed at later stages by the loss of specific cell populations, structural disorganization and atrophy [10,12,13]. Secondary lymphoid follicles become rare or absent [8]. The process of white pulp disorganization is associated with parasite burden, T lymphocyte apoptosis, decreased follicular dendritic cell counts and CXCL13 expression in the spleen, and severe clinical disease [14,15,16,17]. Additionally, increased numbers of plasma cells have also been reported in the spleen and other organs [8,12,18]. In chronic autoimmune diseases, such as systemic lupus erythematous, the permanence of long-lived plasma cells specific for some antigens has a determinant role in the maintenance of this disease [19]. Recent data have shown that plasma cells may also be an important source of IL-10, a cytokine involved in susceptibility to VL [20]. Although we know little regarding how plasma cell accumulation contributes to the progression of VL, the presence of white pulp disruption together with plasmacytosis evidences profound changes in lymphocyte differentiation within the spleen [12,17].

In the primary immune response, stimulated B-cells undergo extrafollicular plasmablast differentiation, becoming short-lived IgM-or IgG-secreting plasma cells with limited lifespan in the medullar cords of lymph nodes and splenic red pulp (reviewed by Tangye, 2011). T- cell dependent antigens also induce B cells to enter follicular germinal centers, where they undergo somatic mutation and antibody class switching, thereby transforming into long-lived plasma cells. These cells preferentially migrate to the bone marrow where they find a restricted number of proper niches that sustain their development [21]. These bone marrow plasma cell survival niches are established by cells capable of producing CXCL12 and IL-6, as well as A Proliferation-Inducing Ligand (APRIL) and B-cell Activating Factor (BAFF) [22]. Under normal conditions, small numbers of short-lived and relatively few long-lived plasma cells are also found in the spleen [23]. In VL, however, the number of these cells progressively increases in the spleen and appears to remain increased despite white pulp atrophy and the absence of secondary lymphoid follicles [8].

In our previous studies, we identified splenic immuno-inflammatory patterns associated with natural infection by *L*. *infantum*. The animals, which evidenced a loss of lymphoid follicle structure and associated cells, perisplenitis and high parasite density in the spleen (in which parasites were detected by conventional histology), were at a more advanced stage of the clinical disease [8,14,16,17]. We therefore hypothesized that this splenic white pulp disruption leads to an abnormal differentiation and homing of B lymphocytes, resulting in a significant accumulation of plasma cells in the spleen. These changes in splenic structure may interfere with the host response to *Leishmania* and impair the spleen’s role in the surveillance against blood borne pathogens, thereby contributing to the progression of VL in addition to favoring the appearance of coinfections. The present study investigated some of the factors associated with plasma cell accumulation that persists in the spleens of dogs with VL even after white pulp disorganization.

We examined the proportion of plasma cells that underwent antibody class switching and the distribution of these cells in the different compartments of the white pulp, as well as in splenic red pulp. We also analyzed the expression of CXCL12, IL-6, APRIL and BAFF, which are the cytokines responsible for organizing plasma cell survival niches capable of prolonging the lifespan of these plasma cells.

## Materials and Methods

### Ethics statement

This study was carried out in strict accordance with the recommendations of Brazilian Federal Law on Animal Experimentation (Law 11794) (http://www.planalto.gov.br/ccivil_03/_ato2007-2010/2008/lei/l11794.htm) and with the Brazilian Health Ministry’s manual for the surveillance and control of VL [24]. This study was approved by the Institutional Review Board for Animal Research (Comissão de Ética no Uso de Animais–CEUA, CPqGM-FIOCRUZ, http://www.bahia.fiocruz.br/?area=01X04, protocol 004/2013).

### Animal samples

A total of 37 canine spleen samples were selected based upon levels of splenic white pulp organization (see below) and positivity for *Leishmania* in spleen cultures. All specimens were obtained from the canine leishmaniasis tissue bank of the Laboratory of Pathology and Bio-Intervention at the Gonçalo Moniz Research Center at Fiocruz-BA in Salvador, Brazil. Samples were obtained from stray dogs of varying breeds and ages collected from the streets of Jequié, BA, Brazil (an endemic area for visceral leishmaniasis) between 2004 and 2010. This was done in collaboration with the Endemic Diseases Surveillance Program of the Bahia State Health Service as part of a program for the surveillance and control of VL. The presence of anti-*Leishmania* antibodies in canine sera was determined by ELISA. Dogs were then sedated with acepromazine (0.1 mg/kg iv, Acepram 1%, Vetnil, Brazil) and sodium thiopental (15 mg/kg iv, Thiopentax 1 g, Cristália, Brazil) and euthanized using a saturated solution of potassium chloride (2 mL/kg, iv). Immediately following euthanasia, splenic aspirates were collected for culturing, and spleen fragments were collected and frozen in liquid nitrogen for molecular biology studies, or were fixed in formalin and embedded in paraffin for morphological studies.

### Clinical data

All animals were subjected to a complete clinical examination, with emphasis given to clinical parameters considered indicative of canine visceral leishmaniasis using criteria previously defined by Lima and colleagues (2014) [17]: Alopecia—Hairless regions in different areas of the skin. Anemia—Pale (light pink) eyes and mouth mucous membranes. Conjunctivitis—Inflammation of the conjunctiva indicated by redness or pus. Dehydration—A poor skin turgor test and/or a delayed capillary refill time. Dermatitis—Dry, exfoliative inflammation of the skin. Emaciation—Skinny, animals with ribs and pelvis evident; Cachectic, animals with ribs and pelvis evident and muscle atrophy in the temporal and scapular regions. Erosion—Presence of shallow, moist, or crusted lesions caused by a loss of the epidermis. Lymphadenopathy—Lymph nodes larger than expected for the size of the animal, as follows: dogs weighing less than 5 kg with lymph nodes larger than 2 cm (large axis), dogs weighing 5–10 kg with lymph nodes larger than 3.5 cm, and dogs weighing more than 10 kg with lymph nodes larger than 4 cm. Onychogryphosis–Nails >1 cm on all extremities. Pustules—Small (2–5 mm) skin vesicles containing pus. Splenomegaly—Animals with a palpable spleen beyond the last rib. Ulcerations—Skin lesions with loss of the epithelial layer, with a granular surface and raised edges. The animals were grouped into the following categories according to the reported clinical signs suggestive of VL: asymptomatic (with none of the clinical signs described above) and symptomatic (with any of the clinical signs described above).

### Anti-*Leishmania* antibody activity in the serum

This was measured by ELISA as previously described [25]. Briefly, 96-well plates were sensitized with crude antigen obtained from *L*. *amazonensis* or *L*. *chagasi*. The plates were washed, blocked with PBS containing 10% of skimmed milk, and the serum of each animal was applied at the dilution of 1:400, followed by an anti-dog total IgG peroxidase conjugate (Sigma). The enzymatic reaction was developed with tetramethyl benzidine (Sigma). A cutoff was established using serum samples obtained from healthy animals from non-endemic areas for visceral leishmaniasis. Values greater than the mean plus three S.D. values of the results obtained from these healthy dogs were considered positive.

### Parasite isolation in cultures

Immediately following euthanization, splenic samples were collected from each animal by puncture using an 18 G x 38 mm gauge needle connected to a 20-mL syringe. Spleen aspirates were cultured in biphasic agar–blood-Schneider’s medium, supplemented with 10% fetal bovine serum as previously described [26]. Cultures were examined weekly for identification of promastigotes, and examinations continued for a period lasting up to 2 months if they remained negative.

### Spleen samples and histological analysis

Spleen tissue slices with a thickness of 3 to 4 mm were cut transversely in relation to the longer axis of the organ. Tissue slices were fixed in formalin and embedded in paraffin. Hematoxylin-and eosin-stained splenic tissue sections with a thickness of 4 to 5 μm were examined by optical microscopy. The samples were examined by two pathologists (LARF and WLCS) and classified into three categories (spleen type 1, type 2, and type 3) according to the degree of structural organization of the splenic white pulp using criteria previously described by Santana and colleagues (2008) [8]. Briefly: spleen type 1 (with well-organized white pulp) had a easily discernible peri-arteriolar lymphocyte sheath, germinal center, mantle zone and marginal zone; spleen type 2 (with slightly disorganized white pulp) had either hyperplastic or hypoplastic changes leading to a loss in definition of the boundaries between the white pulp regions; spleen type 3 (with moderately to extensively disorganized white pulp) had white pulp evident, but with poorly discernible or indistinct regions, or in which the follicular structure was barely distinct from the red pulp and T-cell areas. The latter category is frequently presented with lymphoid atrophy [8]. Since the changes in morphology were heterogeneous in the type 2 spleens, sometimes with mixed patterns of type 1 and type 3, the animals in this category were excluded from the study. Therefore, only animals with clearly organized (spleen type 1) or clearly disorganized (spleen type 3) splenic white pulp were included in the analysis. All animals were organized into three groups based upon the level of splenic white pulp organization and the presence of active infection (defined by either a positive ELISA result or spleen culture): TYPE1SC-: dogs with type 1 spleen and negative serology or spleen culture; TYPE1SC+: dogs with type 1 spleen and positive serology or spleen culture; TYPE3SC+: dogs with type 3 spleen and positive serology or spleen culture.

### Immunophenotyping of spleen cells

Immunophenotyping was performed by immunohistochemistry [27]. Serial spleen sections were placed on poly-L-lysine (Sigma-Aldrich, St. Louis MO, United States)-coated slides and dewaxed. The sections were deparaffinized in xylene, rehydrated in alcohol and any brown formaldehyde pigment was then removed by washing in ammonium hydroxide (10%) in 70% ethanol for 15 minutes. Endogenous peroxidase was blocked using hydrogen peroxide (0.5%) in methanol for 30 minutes. Antigen retrieval was performed with a 0.1% calcium trypsin for 30 minutes. The slides were incubated for 30 minutes with 5% rabbit serum diluted in PBS (phosphate buffered saline containing 15mM NaCl and 10 mM sodium phosphate, pH 7.2) to block unspecific reactions. The slides were then incubated for 30 minutes at room temperature with primary Goat anti-dog-IgA (Fc specific, diluted 1/400), Goat anti-dog-IgM (Fc specific, diluted 1/400) and Goat anti-dog-IgG (Fc specific, diluted 1/200) polyclonal antibodies (Nordic Immunological Laboratories Susteren, Netherlands). As a negative control, sections were incubated with buffer only or with an immunoglobulin of the same isotype of a specificity different from that of the tested antibody. All slides were finally washed with PBS and then incubated for 30 minutes with rabbit anti-goat peroxidase-conjugated antibody (Sigma-Aldrich, St. Louis, MO United State). After peroxidase reactions were developed, all sections were counterstained with hematoxylin, and immunolabeling was evaluated microscopically.

### Morphometry

Images of spleen sections were stained with different antibodies, then captured at a resolution of 1,280 x 1,024 pixels using an Evolution LC digital video camera system (Media Cybernetics, Rockville, MD, United States) coupled to an optical microscope (CX41, Olympus, Japan). Image-Pro Plus version 6.0 software (Media Cybernetics, Rockville, MD, United States) was used to morphometrically estimate red pulp, white pulp and various white pulp compartments, including the periarteriolar lymphocyte sheath (PALS), lymphoid follicles and marginal zones (MZ), in accordance with a previously described procedure [14]. Briefly, the different regions of spleen white pulp were identified according histological descriptions of splenic structure [2] and manually delineated by the observers (JSS and TCS) using a digitizing table and a stylus pen (Wacom—Intuos5—PTH450) using hematoxylin and eosin-stained sections. For the cell counts, random and non-overlapping areas in the red pulp and white pulp compartments were selected. To minimize error for the measurements of the sizes of the white pulp compartments, the following criteria were used: (1) the regions associated with the five largest follicles in each section were selected for analysis of lymphoid follicles and marginal zone, and (2) only arterioles represented in transversal sections were used for PALS analysis. Serial sections of at least one representative slice of each dog’s spleen were used in this analysis [14]. Mean measurements obtained from each of these five compartments were used to further estimate cell population density (number of cells/defined area) in each compartment.

### Cytokine expression analysis by real-time RT-PCR

Splenic expression of CXCR4 and the cytokines BAFF, APRIL, IL6 and CXCL12 was determined using real time RT-PCR. Frozen spleen fragments were thawed and macerated for RNA extraction using the RNeasy Kit (Qiagen, Hilden, Germany) and RNase-free DNase according to manufacturer recommendations. After complementary DNA (cDNA) synthesis using Superscript II reverse transcriptase (Invitrogen Life Technologies, Carlsbad, California, United States), real-time PCR was performed using 10 μl of SYBR1 Green PCR Master Mix (Applied Biosystems, Carlsbad, CA, United States), 200 nM of each primer, 5 μl of cDNA and nuclease-free water was added to achieve a final reaction volume of 20 μl. For each gene of interest, the reaction was performed in duplicate with triplicate negative controls on each plate. The primer pair sequences and the thermal cycle conditions for each gene amplification are shown in Table 1. Cycle threshold values were calculated for each sample. Triplicate standard curves were constructed based on serial dilutions (1:5) using a concentrated pool of cDNA samples as previously described [28]. All reactions were performed in optical 96-well reaction plates using the ABI Prism 7500 system (Applied Biosystems, United States). Cytokine mRNA concentrations for each sample were calculated based on the standard curve, and then normalized using the housekeeping gene, 18S rRNA.

**Table 1:** Primer sequences and annealing temperatures used in real time PCR to detect cytokine and cytokine receptor expression in the spleens of dogs with visceral leishmaniasis.

Gene	Forward primer (5’-3’)	Reverse primer (5’-3’)	Annealing temperature (°C)
18S	CACGGCCGGTACAGTGAAAC	CCCGTCGGCATGTATTAGCT	60°
APRIL	CTTTCACCATGGGTCAGGTGGTGTC	GACACTCAGAATATCGCCCTGGTGC	60°
BAFF	TCTTTGGGGATGAACTGAGC	CAGAAGCTTCAATGCACCAA	55°
IL6	GGCTACTGCTTTCCCTACCC	TTTTCTGCCAGTGCCTCTTT	55°
CXCR4	CAGTTGAGGCTGTGGCAAAC	GAGAGCAGGTATCCAGACGC	55°
CXCL12	TCTTCGAGAGCCACATTGCC	GGGTCAATGCACACCTGTCTG	60°

### Real-time PCR for the detection of *Leishmania* DNA

To detect parasite DNA in frozen spleen samples, a quantitative real-time PCR technique was used [29]. DNA was extracted from each sample using a DNeasy^®^ Blood & Tissue Kit (Qiagen, Hilden, Germany) according to the manufacturer’s protocols. A total of 10 mg of spleen tissue was processed in accordance with Qiagen’s animal tissue protocol. Once extracted, the quality and concentration of each DNA sample was determined using a digital spectrophotometer (NanoDrop^®^ ND-1000, Thermo Scientific, Wilmington, United States). DNA samples were then adjusted to a concentration of 30 ng/μL, aliquoted, and stored at −20°C until time of use. PCR assays were performed using a previously described amplification procedure [30]. The PCR technique targeted a conserved region of *L*. *infantum* kDNA to obtain a 120-bp amplicon. The reactions were performed at a final volume of 25 μL, containing 20 μL of PCR mixture and 5 μL of 30 ng/μL of each DNA sample diluted in deionized water. The PCR mixture consisted of 12.5 μL of Universal Mastermix (Perkin-Elmer Applied Biosystems, Carlsbad, CA, Unites States), 900 nM of each forward primer 5'-AACTTTTCTGGTCCTCCGGGTAG-3' (LEISH-1) and reverse primer 5’-ACCCCCAGTTTCCCGCC-3' (LEISH-2), and a fluorogenic probe (5’-AAAAATGGGTGCAGAAAT-3’), which was synthesized using a FAM reporter molecule attached to the 5′ end and a MGB-NFQ quencher linked to the 3′-end (Perkin-Elmer Applied Biosystems, Carlsbad, CA, United States), at a concentration of 200 nM. A standard curve was generated using serial dilutions performed in triplicate of *L*. *infantum* DNA from 10^6^ to 10^−1^ parasites/mL. The amplifications were performed in triplicate for each sample, as well as negative controls, using an ABI Prism 5900 sequence detection system (Perkin-Elmer Applied Biosystems, Carlsbad, CA, United States). The standard curve was generated by plotting the Ct values against the standardized parasite concentrations. The cycling parameters were 50°C for 2 min, 95°C for 10 min, 40 cycles at 95°C for 15 s, and 60°C for 1 min. A canine housekeeping gene (18S rRNA) was amplified to normalize the concentration of the input sample DNA, as well as to ensure that negative results were not attributable to sample inadequacies, including the absence of host cells in aspirated samples, DNA degradation, low amounts of loaded DNA, or the presence of PCR inhibitors in samples. Pre-developed TaqMan assay reagents (Perkin-Elmer Applied Biosystems, Carlsbad, CA, United States) were used to amplify the 18S rRNA gene as an internal reference for canine genomic DNA. All reactions were performed at a final volume of 25 μL, containing 20 μL of PCR mixture and 5 μL of 30 ng/μL of each canine DNA sample diluted in deionized water. The PCR mixture consisted of 12.5 μL of Universal Mastermix (Perkin-Elmer Applied Biosystems, Carlsbad, CA, United States) and 1.25 μL of 18S GeneEx Assay (Perkin-Elmer Applied Biosystems, Carlsbad, CA, United States) at a concentration of 20x. Deionized water was added to a final volume of 25 μL. Standard curves ranging from 450 to 18.75 ng of the housekeeping gene were prepared, with all dilutions performed in triplicate on each plate. Amplification reactions were also performed in triplicate for each sample using an ABI Prism 5900 sequence detection system (Perkin-Elmer Applied Biosystems, Carlsbad, CA, United States). The cycling parameters were 50°C for 2 min, 95°C for 10 min, and 40 cycles at 95°C for 15 s and 60°C for 1 min. For each sample, the amount of target and housekeeping DNA was determined by comparing the obtained Ct values with the appropriate standard curve. Parasite load was expressed as the number of parasites normalized to the established reference amplification value for the 18S rRNA housekeeping gene in 100 mg of host tissue.

### Biochemical analyses

Blood samples obtained for serum biochemical analyses were collected from the cephalic veins of dogs under manual restraint. Serum collected by centrifugation after coagulation in Vacutainer^®^ tubes was used for biochemical tests to quantify total protein, albumin and globulin by an enzymatic colorimetric method using an A15 auto-analyzer (BioSystems, Barcelona, Spain).

### Electrophoretic Analysis of Serum Proteins

Electrophoretic canine serum analysis allowed for the evaluation of concentrations of albumin, alpha-1, alpha-2, beta and gamma globulin. Agarose gel was used according to the procedure described by the manufacturer (Celmgel, Celm^®^. São Paulo, Brazil). Samples were electrophoresed for 28 min at 100 volts, then fixed and stained in Amido black solution, followed by destaining in acetic acid. Electrophoretic analysis of the serum protein fractions was performed using SDS—60 (SE250 Electrophoresis System—CELM) software. Sera from healthy dogs (from a non-endemic area) were used as controls in electrophoretic analysis.

### Enzyme immunoassay (ELISA)

To investigate the presence of polyclonal B cell activation, sera from dogs with VL were tested by ELISA to detect the concentration of IgG antibodies reactive to two different antigens: KLH (Keyhole limpet hemocyanine) and OVA (Ovalbumin). Briefly, these antigens were diluted to 10 μg/mL in coating buffer (15 mM Na_2_HCO_3_, 28 mM NaHCO_3_, pH 9.6), placed into wells on 96-well microtiter plates, incubated overnight at 4°C. Next, non-specific reactions were blocked with PBS, pH 7.2, containing 0.1% Tween 20 and 0.5% gelatin. The wells were then reincubated with canine serum at a dilution of 1:100 in PBS-gelatin. These dilutions had been shown to constitute the best serum concentrations to produce less false-negative and false-positive results. Wells were then washed with PBS, pH 7.2, containing 0.05% Tween 20. Peroxidase-conjugated rabbit anti-dog IgG (diluted 1:10,000) (Sigma-Aldrich, St. Louis, MO, United States) was added and the plates were incubated for one hour at 37**°**C. Reactions were developed with tetramethylbenzidine (Sigma-Aldrich, St. Louis, MO, United States) and H_2_O_2_ in 0.1 M acetate-citrate buffer, pH 5.0, then stopped with H_2_SO_4_ and read in a spectrophotometer with a 450 nm filter (Emax Precison Microplate Reader, Molecular Devices Corporation, Sunnyvale, CA, USA).

### Expression and Statistical Analysis

Numerical data representing absolute values, means, medians or proportions are shown in tables and graphs. The Kruskal-Wallis test was used for comparisons of absolute values involving more than two groups. For comparisons involving proportions, the Chi-square test with the Yates’ correction or Fisher’s exact probability test were used. Correlations between variables were studied using the Spearman test [31]. The level of significance was established at P < 0.05.

## Results

### General characteristics of the animals

The main characteristics of the animal groups used in this study are summarized in Table 2. Although spleen aspirate cultures and serology were negative in the TYPE1SC- group, PCR results for *Leishmania* detection were positive in 5 out of the 9 animals of this group. The number of clinical signs associated with VL and parasite load in the spleen was higher in the TYPE3SC+ group (4.5 [3.0–6.8] and 2.09 [0.09–116.80], median and interquartile range, respectively) than in the TYPE1SC- group (2.0 [0.0–4.0] and 0.02 [0.00–0.86] respectively).

**Table 2:** General characteristics of included non-infected dogs, dogs without active infection with organized white pulp (TYPE1SC), infected dogs with organized white pulp (TYPE1SC+) and infected animals with disorganized white pulp (TYPE3SC+).

PARAMETERS	TYPE1SC-		TYPE1SC+		TYPE3SC+	
*N*	9		16		12	
Gender (male:female)	6:3		10:6		6:6	
Positive test for *Leishmania* infection:						
Spleen culture (%)	0	(0)	10	(62)	9	(75)
Serology (%)	0	(0)	16	(100)	11	(92)
PCR (%)	5	(56)	15	(94)	10	(83)
Parasite/100 mg of spleen*	0.02	[0.00–0.86]^a^	0.10	[0.01–4.44]	2.09	[0.09–116.80]^a^
Clinical signs of VL:						
Symptomatic (%)	6/9	(66)	15/16	(94)	12/12	(100)
Number of clinical signs of VL*	2.0	[0.0–4.0]^a^	3.0	[2.0–3.0]	4.5	[3.0–6.8]^a^

*Median [interquartile range].

^a^ = Statistically significant difference Kruskall-Wallis, ^a^ = P<0.05.

### Plasmacytosis and lymphoid disruption in canine visceral leishmaniasis

**Fig 1:**
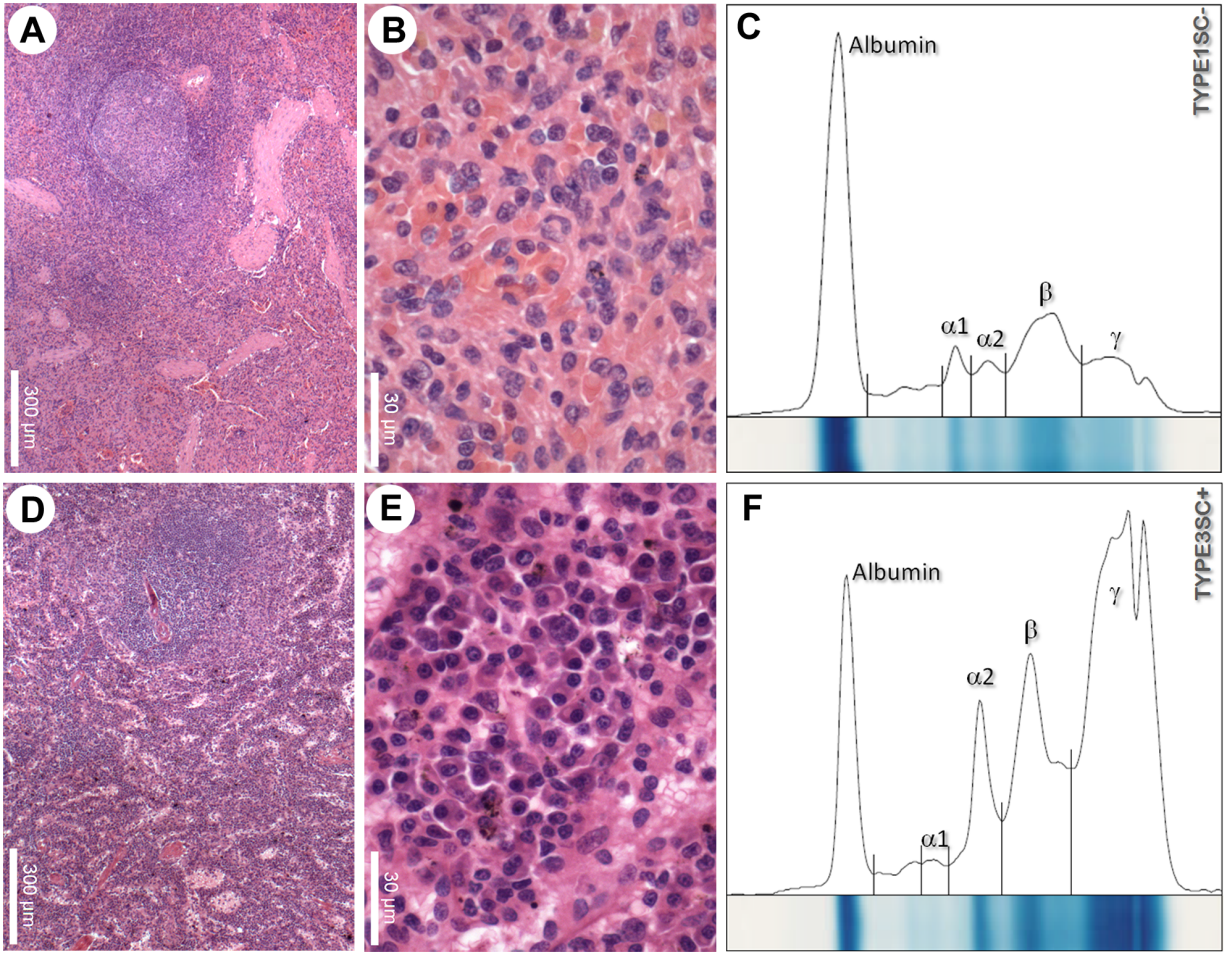
Plasmacytosis and dysproteinemia in dogs with visceral leishmaniasis and disruption of splenic white pulp. A-B–normal spleen of control TYPE1SC- animals. A—well-organized white pulp with secondary lymphoid follicles and distinct marginal and periarteriolar zones. B–red pulp with varied cell populations. C–normal pattern of serum protein distribution in electrophoresis. D-E–spleen with white pulp disruption in TYPE3SC+ animals. D–barely evident primary lymphoid follicle and inconspicuous marginal zone. E–red pulp plasmacytosis and (F) serum protein electrophoresis with a polyclonal pattern of gamma globulin distribution.

To study the association between plasmacytosis and the disruption of splenic lymphoid tissue, we semiquantitatively estimated the frequency of plasma cells in the spleens of the animals from each group. A trend towards an increased frequency of plasma cells in the spleens of animals with active infection was further accentuated in those also exhibiting white pulp disorganization (Table 3 and Fig 1). Plasmacytosis was seen in most of the animals in the TYPE3SC+ group (8/11), with moderate or intense plasmacytosis evidenced in six, a proportion higher than in the other groups (Fisher test, P<0.05).

**Table 3:** Laboratory findings of included non-infected dogs, dogs without active infection with organized white pulp (TYPE1SC), infected dogs with organized white pulp (TYPE1SC+) and infected animals with disorganized white pulp (TYPE3SC+).

PARAMETERS	TYPE1SC-		TYPE1SC+		TYPE3SC+	
Plasmacytosis:						
Absent	5	(56%)	8	(50%)	4	(33%)
Slight	3	(33%)	5	(31%)	2	(17%)
Moderate	1	(11%)	3	(19%)	4	(33%)
Intense	0	(0%)	0	(0%)	2	(17%)
Serum protein pattern:*						
Total protein (g/dL)	6.5	[5.6–8.5]	7.3	[7.1–8.7]	8.3	[6.5–8.6]
Albumin (g/dL)	2.5	[2.1–3.0]	2.7	[2.1–3.3]^a^	1.9	[1.5–2.4]^a^
Globulin (g/dL)	4.3	[3.4–5.4]	5.0	[4.1–6.4]	6.2	[4.1–6.9]
Albumin/globulin ratio	0.6	[0.5–0.7]^a^	0.6	[0.4–0.7]^b^	0.3	[0.3–0.4]^a^^,^^b^
Serum protein electrophoresis:*						
Albumin (%)	42.0	[31.1–47.0]^b^	35.8	[27.2–46.4]	20.9	[17.2–28.0]^b^
Alpha-1 (%)	5.6	[4.0–7.3]^b^	4.7	[4.2–5.0]	3.2	[2.6–4.2]^b^
Alpha-2 (%)	5.3	[3.0–8.0]	5.2	[4.9–7.7]	3.9	[1.7–6.5]
Beta (%)	28.6	[27.5–34.0]	28.2	[23.4–30.2]	26.2	[24.0–38.2]
Gamma (%)	16.4	[15.0–26.2]^b^	26.0	[19.0–35.0]	42.5	[28.2–49.2]^b^
Anti-*Leishmania* ELISA	0.88	[0.74–1.34]^b^	1.30	[1.24–2.21]	2.47	[1.62–2.84]^b^
ELISA with unrelated antigens:*						
Ovalbumin (OD 450 nm)	0.20	[0.18–0.45]	0.40	[0.40–0.35]	0.39	[0.29–0.39]
Keyhole limpet hemocyanin (OD 450 nm)	0.42	[0.27–0.60]	0.50	[0.33–0.58]	0.64	[0.49–0.80]

*Median [p25-p75].

^a or b^ = Statistically significant difference Kruskall-Wallis, ^a^ = P<0.05, ^b^ = P<0.01.

### Dysproteinemia in canine visceral leishmaniasis

Splenic plasmacytosis was observed to correlate with the changes observed in the patterns of serum protein distribution among the animal groups. Median values of serum concentrations of total protein and of globulin from groups TYPE1SC+ (7.3 [7.1–9.7] g/dL and 5.0 [4.1–6.4] g/dL respectively) and TYPE3SC+ (8.3 [6.5–8.6] g/dL and 6.2 [4.1–6.9] g/dL respectively) were above the reference values for normal dogs (5.4–7.1 g/dL for total protein and 2.4–4.4 g/dL for globulin, http://www.rkdiagnostico.com.br/tabela_valores.pdf). Conversely, the median of serum concentration of albumin in most animals of group TYPE3SC+ (1.9 [1.5–2.4] g/dL) was below the reference values (2.6–3.3 g/dL). Furthermore, the albumin/globulin ratio was lower in the TYPE3SC+ group (0.3 [0.3–0.4]) than in the TYPE1SC- (0.6 [0.5–0.7], P<0.05) or TYPE1SC+ groups (0.6 [0.4–0.7], P<0.01, Kruskall-Wallis test, Table 3).

### Serum protein electrophoresis

In order to confirm that increased globulin levels observed in the animals of group TYPE3SC+ were mostly due to the presence of antibodies, electrophoresis of serum proteins was performed. As expected, the albumin region size was smaller in the animals of the TYPE3SC+ group (20.9 [17.2–28.0]%) than that of the TYPE1SC- (42.0 [31.1–47.0]%, ANOVA, P<0.01) group. Alpha 1 protein expression was also diminished in the TYPE3SC+ (3.2 [2.6–4.2]%) animals in comparison with the TYPE1SC- (5.6 [4.0–7.3]%, ANOVA, P<0.01) group (Table 3). An expressive increase in gamma globulin was observed in the TYPE3SC+ (42.5 [28.2–49.2]%) group in comparison to the TYPE1SC- (16.4 [15.0–26.2]%, Kruskall-Wallis test, P<0.01) group (Table 3). Additionally, the gamma globulin region was wide, reflecting the presence of polyclonal immunoglobulin (Fig 1F).

### Plasmacytosis and serum dysproteinemia

To determine the extent to which splenic plasmacytosis was associated with alterations in the spectrum of serum proteins, correlations between the intensity of spleen plasmacytosis and changes in serum parameters were investigated. Spleen plasmacytosis was found to be directly associated with serum concentrations of globulin (Spearman r = 0.568, 95%CI 0.285 to 0.760, P<0.001), gamma globulin fraction (Spearman r = 0.609, CI 0.302 to 0.802, P<0.001) and inversely associated with the albumin/globulin ratio (Spearman r = -0.684, CI -0.830 to -0.450, P<0.001).

### Polyclonal B-cell activation and serum antibody activity against antigens unrelated to *Leishmania*

To determine the contribution of non-specific immunoglobulins to increased gamma globulin concentrations, serum samples were examined by ELISA for antibodies reactive to OVA and KLH, antigens to which the animals presumably had not been previously exposed. Progressively higher optical density values were observed in groups TYPE1SC-, TYPE1SC+ and TYPE3SC+, corresponding to the detection of antibodies reactive to OVA or KLH, yet without statistical significance (Table 3).

### Immunoglobulin isotypes associated with splenic plasmacytosis

**Fig 2:**
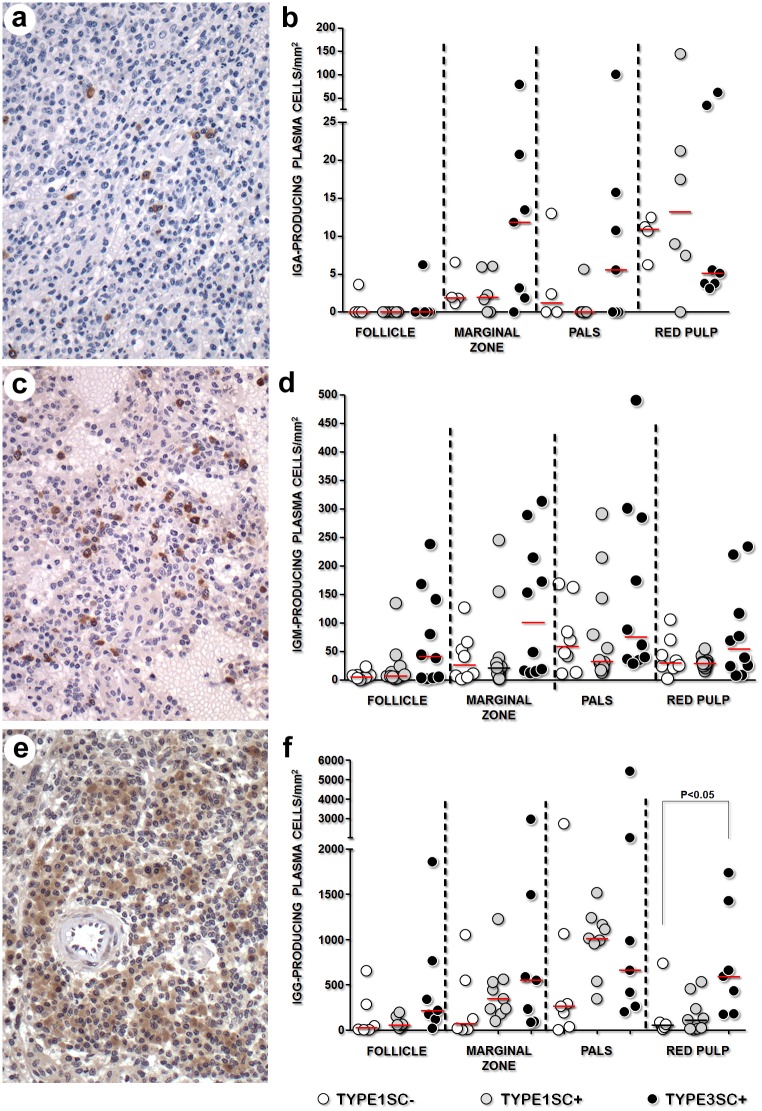
Isotype of the immunoglobulins produced by plasma cells in dogs with normal spleens and inactive (TYPE1SC-) or active (TYPE1SC+) *Leishmania* infection, or those with disorganized white pulp and active *Leishmania* infection (TYPE3SC+). (Kruskall-Wallis test). **a** and **b** correspond to IgA; **c** and **d** correspond to IgM and **e** and **f** correspond to IgG.

To better characterize the plasma cell populations that accumulate in the spleen over the course of VL, the immunoglobulin isotypes produced by these cells were examined. IgG-secreting cells were the predominant plasma cells present in the spleens of all animals, regardless of study group. These cells were preferentially observed around periarteriolar areas and in large aggregates within red pulp. In some TYPE3SC+ dogs, IgG+ plasma cells were also seen in lymphoid follicles. IgM-secreting plasma cells were present in the marginal zones and in periarteriolar areas. In the red pulp, IgM-secreting plasma cells formed discrete small aggregates. IgA-secreting plasma cells were rare, with no defined distribution observed (Fig 2). Although a trend towards an increased density of plasma cells bearing any of the immunoglobulin isotypes was observed in the TYPE3SC+ group, a significantly increased density of the IgG+ plasma cell isotype was exclusively observed in the red pulp of the TYPE3SC+ (582 [178–1429] cells/mm^2^) group, in comparison to the control TYPE1SC- group (54 [26–249] cells/mm^2^, Kruskal-Wallis, p<0.05, Fig 2).

### Cytokine expression in the spleens of dogs with visceral leishmaniasis

**Fig 3:**
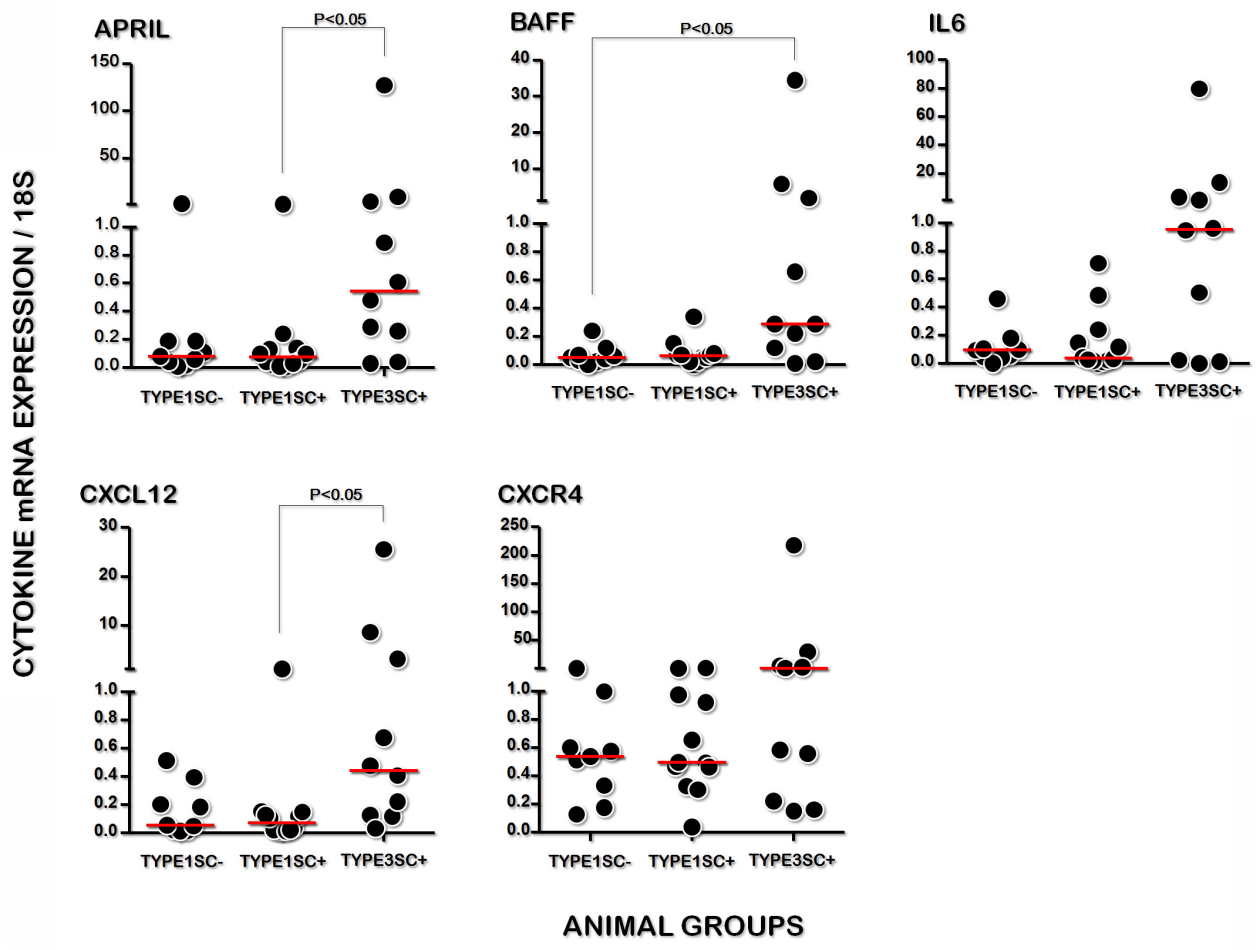
Gene expression of APRIL, BAFF, IL6, CXCL12 and of the CXCR4 chemokine receptor in the spleens of normal dogs, those with inactive (TYPE1SC-) or active (TYPE1SC+) *Leishmania* infection, or those with disorganized white pulp and active *Leishmania* infection (TYPE3SC+). (Kruskall-Wallis test).

To investigate the determinants of plasma cell accumulation in the spleen, we quantified the gene expression of cytokines associated with the survival and permanence of these cells in splenic tissue. Higher gene expression of APRIL and CXCL12 was found in the splenic tissue of group TYPE3SC+ (0.54 [0.20–5.42] and 0.44 [0.12–4.53] relative to 18S expression, respectively) than in the animals of TYPE1SC+ (0.08 [0.03–0.19], P<0.05 and 0.07 [0.02–0.14], P<0.01, relative to 18S expression, respectively, Kruskal-Wallis test, Fig 3). BAFF expression was found to be higher in the spleens of TYPE3SC+ animals (0.29 [0.10–2.88]) than in the animals of TYPE1SC- (0.05 [0.02–0.10], both relative to 18S expression, Kruskal-Wallis test, P <0.05. Fig 3).

## Discussion

The present study clearly demonstrates that, in severe canine VL, the disruption of splenic white pulp is associated with more frequent and intense plasma cell accumulation in the spleen. The group of animals with active infection and white pulp disorganization presented with more severe dysproteinemia and increased globulin fraction in the serum, which also correlated with the intensity of plasma cell accumulation. IgG-producing cells were predominantly responsible for this increased plasma cell density in the spleen. In addition, the CXCL12, APRIL and BAFF cytokines, which are associated with plasma cell homing and survival [21], were more highly expressed in the splenic tissue of animals with active infection and white pulp disruption.

In VL, plasmacytosis is striking and frequently associated with pronounced serum dysproteinemia [32]. Although routine testing for dysproteinemia has always been common in the diagnosis of VL [24], little is known regarding the genesis and potential implications of plasma cell accumulation in the course of this disease. Under normal conditions, plasma cells transiently accumulate in the lymphoid organs after a brief course of stimulation by certain antigens [21]. Most of these cells are short-lived IgM- or IgG- secreting cells [33]. Although splenic red pulp may also support the survival of a small number of long-lived plasma cells, most of the plasmablasts that give rise to these long-lived plasma cells then migrate to the bone marrow where they find survival niches [21]. Interestingly, in the studied dogs with active infection, plasma cells were observed to accumulate in the spleen, even when disorganization and atrophy of lymphoid follicles occurred. This finding suggests that many of these plasma cells may differentiated extrafollicularly. Lymphoid follicle disruption and plasmacytosis has been reported in experimental *Plasmodium* infection [34]. Additionally, the massive plasma cell accumulation in the spleens of animals with extensive white pulp disorganization suggested these cells’ extended life-span.

In fact, the differentiation of long-lived plasma cells may be induced in the secondary lymphoid organs under conditions of chronic inflammation, such as chronic visceral leishmaniasis [19]. The permanence of these cells in bone marrow and in other tissues requires the existence of specific environmental niches containing cells capable of producing IL-6, BAFF, APRIL and CXCL12, cytokines, which support plasma cell differentiation, homing and survival [22,35,36,37]. BAFF and APRIL are homologs of the TNF superfamily that bind to BCMA and to TACI (BAFF also engages the BAFF receptor), thereby inducing the Bcl-2 anti-apoptotic pathway [23]. CXCL12 binds to the CXCR4 receptor expressed by plasma cells [22]. This chemokine may modulates LFA1 function in splenic plasma cells, which contributes to their homing in splenic red pulp [22]. Although all of these cytokines were expressed in the dogs herein, BAFF, APRIL and CXCL12 were upregulated in the spleens of the animals with active *Leishmania* infection and white pulp disruption. A trend towards increased IL6 and CXCR4 expression was also observed in the animals of the TYPE3SC+ group, yet no statistically significance was detected. Nevertheless, this data on cytokine expression suggests that plasma cell survival niches are generated in the spleen in severe forms of visceral leishmaniasis. Consequently, the plasmacytosis observed in the animals with active infection and disrupted white pulp may be the result of the generation, retention and extended survival of plasma cells in the spleen. Unfortunately, given the field conditions in which this study was performed, it was not possible to identify the actual sources of cytokine production in the spleen. It has been shown that plasma cells are capable of building an autocrine stimulatory loop based on the presence of these cytokines in the lymph nodes of patients with Systemic lupus erythematous [38]. The formal identification of the sources of upregulated cytokines in disrupted spleens in canine VL warrants further study.

Bone marrow presents a restricted number of survival niches for long-lived plasma cell development. Under normal conditions, these niches may correspond to 0.5% of bone marrow cellularity [39]. Chronic inflammatory diseases that course with polyclonal B cell activation, such as visceral leishmaniasis, may lead to a 5–20% increase in plasma cell density in bone marrow [40]. The long-life plasma cells that are unable to find a niche within bone marrow likely recirculate back to the spleen, where they may find a newly generated survival niche due to the inflammatory response.

Recently, it has been suggested that high CD138, increased CD9 and weak CD62L, CD45 and HLA-DR expression may distinguish long-lived plasma cells from short-lived cells and plasmablasts [37]. Furthermore, long-lived-plasma cells from the spleen and bone marrow have been reported to differently express adhesion molecules in [41]. Hence, further studies employing flow cytometry to characterize the plasma cells present in the spleens of animals with severe leishmaniasis may contribute to a better understanding of the underlying mechanism of plasmacytosis in association with leishmaniasis or other chronic inflammatory diseases.

It has been proposed that secondary lymphoid organ plasmacytosis is mainly responsible for the hypergamaglobulinemia associated with chronic inflammatory diseases [19]. In this work we showed the splenic plasmacytosis is strongly associated with high serum globulin concentrations, a relative increase in gamaglobulin fraction in the serum and serum dysproteinemia. Serum protein electrophoresis demonstrated that the hypergamaglobulinemia presented by the animals with infection and white pulp disruption was clearly polyclonally distributed [42]. The study of antibody activity against ovoalbumin and KLM showed no statistically significant differences among the experimental groups. However, a trend towards increased antibody activity against these antigens was observed. Cross-reactivity with antigens unrelated to *Leishmania* has been demonstrated in the sera of humans with visceral leishmaniasis [43].

Our data serves to contribute to the understanding of the disruption of the splenic histological structure and plasmacytosis observed in severe forms of VL, evidenced by marginal zone and lymphoid follicle atrophy, effacement of white pulp compartment boundaries, loss of B-, T- and follicular dendritic cells in spleen compartments and progressive plasma cell accumulation in the spleen [14,15,16]. Such changes in splenic structure may be associated with an impairment in the host capability to respond to infection and, consequently, progression to death [12]. Recently, Goto and colleagues showed an increase in BAFF serum levels in human patients with active VL [44]. In severe VL, the occurrence of these changes in splenic structure, in conjunction with dysproteinemia and increased levels of cytokines in the blood [17] lends support for the inclusion of these parameters in a panel of disease prognosis markers. Further studies are necessary to assess the relationship between increases in levels of BAFF and other cytokines in the blood, as well as their upregulation in the disrupted spleen.
